# Mechanisms of action for digital therapeutics

**DOI:** 10.1038/s41746-026-02502-y

**Published:** 2026-03-05

**Authors:** Linea Schmidt, Benedikt Langenberger, Felix Schirmann, Simon Reif, Ariel Dora Stern

**Affiliations:** 1 Hasso Plattner Institute, Digital Engineering Faculty, University of Potsdam, Potsdam, Germany; 2 Windreich Department of Artificial Intelligence & Human Health, Icahn School of Medicine at Mount Sinai, New York, NY United States of America; 3 Oviva AG, Berlin, Germany; 4 ZEW - Leibniz Centre for European Economic Research, Mannheim, Germany; 5 University of Erlangen-Nürnberg, Nürnberg, Germany

**Keywords:** Business and industry, Drug discovery, Health care, Medical research

## Abstract

Digital therapeutics (DTx) are increasingly established, yet their mechanisms of action (MoA) remain underexplored. This article defines and categorizes DTx MoA through a novel conceptual framework, distinguishing them from conventional treatments. We specifically conceptualize therapeutic elements, cognitive-affective and behavioral changes, and segment the notion of “dose” into distinct categories. This actionable framework provides a systematic basis for enhancing DTx research, design, and clinical effectiveness.

## Introduction

Digital therapeutics (DTx) refer to “health software intended to treat or alleviate a disease, disorder, condition, or injury by generating and delivering a medical intervention that has a demonstrable positive therapeutic impact on a patient’s health”^1^. To achieve their therapeutic effects, DTx leverage the tracking and visualization of health parameters and/or deliver targeted guidance and therapeutic interventions. Most DTx applications are intended to drive some form of cognitive-affective or behavioral change—whether by promoting healthier lifestyle choices, supporting adherence to conventional therapies such as pharmaceuticals, or enhancing the success of medical procedures (e.g., pelvic floor rehabilitation after urological surgery). Others focus on educating patients on their condition, strengthening patients’ self-management and self-efficacy in daily life, or providing supplements or alternatives to traditional therapies and/or therapeutics. Recent years have seen tremendous growth in DTx research and development across a wide range of therapeutic areas^2^.

Although DTx employ a variety of treatment approaches (e.g., improved awareness of symptoms, motivation to self-manage health, cognitive-behavioral therapy) to achieve therapeutic effects, the underlying principles of how they work—their so-called *mechanisms of action* (MoA) are rarely discussed directly^3^. The term MoA originates from the pharmaceutical domain and refers to how a substance–typically a drug–acts on the body to produce a treatment effect^4^. Ribba et al. have drawn parallels between traditional pharmaceuticals and digital therapeutics (DTx), particularly in terms of clinical trial design, the concept of an “active ingredient” necessary to produce a therapeutic effect, and their dosing^3^.

The MoA of DTx can be either direct or indirect. A direct therapeutic effect aims to change behavior, or lifestyle, which in turn directly impacts the desired health measure. An indirect therapeutic effect aims to increase adherence to other interventions, such as medication adherence. DTx can be stand-alone therapies (e.g., most of the German “DiGAs” (Digital Health Applications)^5^ and French PECAN applications)^5^ and are designed to work in conjunction with traditional therapeutic and interventional elements^6,7^, such as prescription medications or surgical procedures. One of their key goals in this context is to support and increase adherence to these conventional treatments, thereby improving clinical outcomes. To better understand and characterize the mechanisms of action for DTx, this article conceptualizes MoA and draws direct comparisons to established therapies and evidence-based therapeutic approaches such as pharmaceuticals, psychotherapy, and physiotherapy.

## General mechanism of action of DTx

**Fig. 1:**
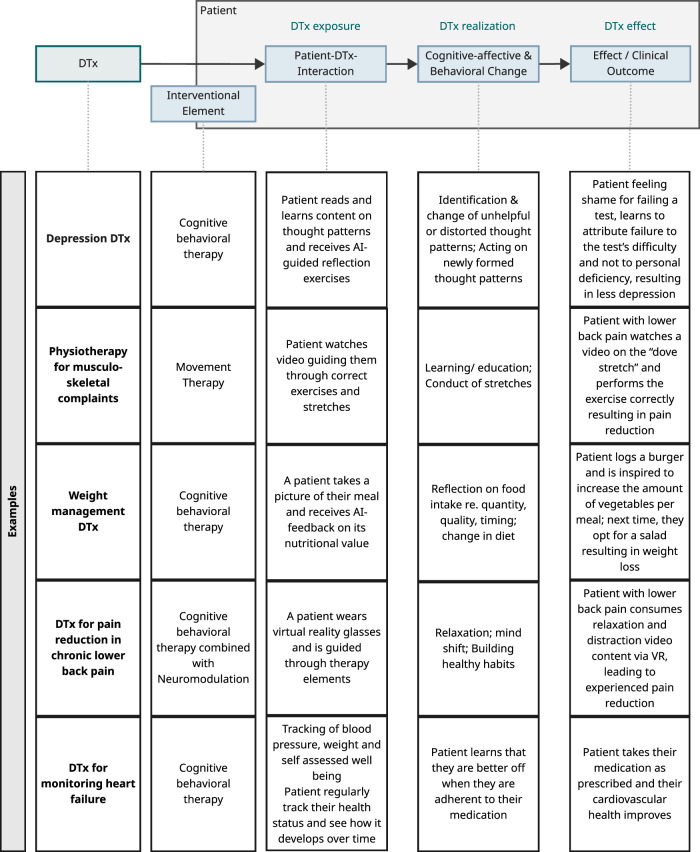
From DTx to clinical outcome. Fictional examples with common DTx intervention types and their intended cognitive-affective and behavior change. The depicted mechanisms are simplified representations and may involve multi-step pathways and interacting processes in practice.

All DTx work by impacting patients’ mental state (“cognitive-affective change”), driving patients’ behaviors, or both. The “therapy” itself is triggered by the interaction of a patient and a DTx, with condition-specific interventional elements leading to the intended treatment effect. The nature of the therapeutic intervention itself depends on the clinical use case and the intended treatment effect, e.g., for neurological and mental health conditions, cognitive behavioral therapy elements are very common (see Fig. 1).

## Implications for measurability

A clear definition of the mechanism of action for a DTx along with operationalization of the interventional elements, patient-DTx interaction, intended cognitive-affective and behavior change (e.g., using behavior change techniques [4]), and the desired clinical outcome is needed to derive testable hypotheses^8^. This may allow more meaningful interpretation of data from studies of digital therapeutics.

Due to the digital nature of DTx, a significant amount of metadata is collected automatically—for example, the number of logins, session duration, and the number of completed questionnaires. In addition to this, clinically relevant outcome data are also collected directly through patient-DTx-interactions, resulting in patient-reported outcome measures (PROMS, which are typically collected via embedded versions of validated questionnaires), vital signs, or images. Measures derived from patient-DTx-interactions, such as timing, duration, frequency, and extent of use, are often operationalized as “patient engagement”^9^. Such real-world data (RWD) can be employed in DTx research and development as well as used in DTx approval and reimbursement. Engagement, however, needs to be meaningful in order to be clinically effective: simple exposure to a DTx (e.g., logging in) is not sufficient to effect changes in behavior and therefore health^10,11^.

**Fig. 2:**
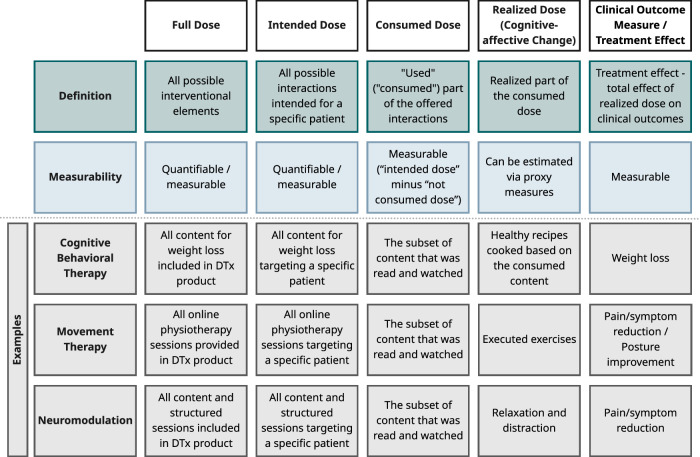
DTx dose definitions. Different definitions of doses in DTx along the path from manufacturer/clinician intended dose through changes in clinical outcomes.

Impactful patient-DTx-interactions are a precondition for clinically meaningful cognitive-affective and behavior change. We refer to the patient-DTx-interactions as “consumed dose”, which may vary per DTx and, additionally, patient. The final measurable change in clinical outcomes, the therapeutic effect, is not (necessarily) a direct function of the “consumed dose” but rather of the actual dose of DTx that is realized by a patient, i.e., that led to a cognitive-affective and behavior change. Examples for these different steps are provided in Fig. 2.

A DTx treatment can be heterogeneous in several ways. The optimal dose may vary from patient to patient (individual-level variation), and even for a single patient, it may change over time (time-dependent variation). Additionally, because DTx may monitor cognitive-affective and behavior changes, the intensity of tracking may evolve throughout the treatment period in context-appropriate ways. For example, many patients benefit from more frequent monitoring and interaction at the start of treatment and require less as progress is made and/or as inputs that impact behavioral change are internalized. Consider a DTx for weight loss that involves meal logging: patients may log their meals consistently at first, but as healthy cooking habits become habitual, the same individuals tend to track less frequently (time-dependent variation)–indeed, the more intensively the behavior change is internalized, the *less* the patient will need to interact with the DTx in the future.

## Comparison of DTx and traditional therapy forms

Many DTx mimic behavioral change interventions that are well-established in physiotherapy or psychotherapy with the intention of modifying the patients’ cognitive-affective or behavior for the long run. These approaches, in turn, demand more patient initiative than receiving and administering a pharmaceutical.

The different paths from dose planning to mechanism of action for pharmaceuticals, physio- and psychotherapy as well as DTx are summarized in Table 1. Compared to traditional therapy elements, the MoA in DTx is non-deterministic and highly patient-mediated, meaning that unlike pharmaceuticals, which are active biological agents that act according to direct biological mechanisms, DTx target cognitive-affective and behavioral changes. While determining the adequate dose for pharmaceuticals is challenging given the various factors that influence effectiveness and potential side-effects^12^, the concept of adequate dosing is even more difficult to assess with behavioral interventions. Since the majority of DTx target behavior change, a comparison to psychotherapy or physiotherapy sessions is often the closest conceptual analog.

**Table 1:** Conceptual overview of differences between traditional therapy forms and DTx

Pharmaceutical administered by HCP	Self-administered Pharmaceutical	Psychotherapy / Physiotherapy	DTx
Dose planning			
Defined and fixed by HCP	Defined and fixed by HCP	Defined and fixed by HCP	Loosely defined but not fixed
Static (changes only as recommended by HCP)	Static (changes can be made by patient–e.g., acute medication only as needed, or by HCP)	Varying, depending on patients’ position in treatment	Varying, depending on patients’ position on the digital treatment pathway
Dose intake			
Controllable	Depends on patient adherence	Controllable	Depends on patient adherence
Dose measurement			
Trackable dose intake	Not trackable dose intake	Trackable dose intake	Partially trackable dose intake via DTx metadata
Realization of therapeutic effect			
Biological: independent of patients’ compliance	Biological: conditional on patients’ compliance	Behavioral: contingent on patients’ compliance	Behavioral: contingent on patient compliance
Mechanism of action			
Intended dose = consumed dose = realized dose → clinical outcome	Intended dose ≥ consumed dose = realized dose → clinical outcome	Intended dose = consumed dose ≥ realized dose → clinical outcome	Intended dose ≥ consumed dose ≥ realized dose → clinical outcome

*HCP* health care professional.

As is the case for sessions of traditional psycho- or physiotherapy, which DTx may (partially) replace or reinforce, for DTx there is typically no exact predefined number of sessions needed, though clinicians might be influenced by reimbursement schemes and discontinuities therein (e.g., no further reimbursement after a pre-specified number of sessions or a pre-specified amount of intervention time). Importantly, DTxare self-administered at the discretion of the patient, throughout the patient journey^12^. Just as pharmaceutical treatments rely on patients consistently taking their medication, the effectiveness of DTx depends on patients’ engagement and compliance with implementing the recommended cognitive-affective and behavior changes.

In the pharmaceutical industry, significant research has been devoted to optimizing drug design to improve adherence. Similarly, usability and engagement optimization are becoming increasingly important in DTx to enhance adherence and thereby potentially effectiveness. In this context, there is significant potential for digital biomarker research and development; better digital biomarkers hold the potential to more accurately quantify individual cognitive-affective and behavior changes^13^. This is particularly promising given the digital nature of DTx and their unique ability to generate RWD, which can be leveraged to derive real-world evidence (RWE)^14,15^.

However, it remains the case that optimal interaction with a DTx is highly individual and cannot be measured solely by the number of logins or time spent using an app. Instead, it reflects the personalized amount and type of therapeutic elements required to achieve the best treatment outcomes as well as the *realized dose*, which is likely also highly personalized, even conditional on intended and consumed doses.

## Implications for DTx research

Given the complexity of the mechanism of action of DTx and the heterogeneity of current research, we propose the following four fundamentals to guide DTx research:

1. Clear MoA definition: Individual DTx should have a clearly-defined MoA from which MoA-specific hypotheses are derived for research purposes. MoA-specific hypotheses should be pre-specified to limit spurious findings from testing many features and outcomes.
2. Specific operationalization: Evaluations should include the operationalization of direct or verifiable interventional elements for patient-DTx-interaction, intended and realized cognitive-affective change and behavior as well as the specific relation to patient-relevant clinical outcomes. When multiple interventional elements and outcomes are analyzed, researchers should adjust for multiplicity and clearly label exploratory analyses.
3. Priority of dose-realization: The actual cognitive-affective or behavioral change is the driver of clinical outcomes (dose realization) in a given individual; the interaction with the DTx (dose intake or “engagement”) is a necessary but not sufficient condition for this. Hence, DTx research should focus on clinically meaningful transformations in patients.
4. Awareness of individual response and dose-heterogeneity: Importantly, dose realization may differ across individuals and over time in the treatment journey; hence, individual, time-sensitive dose finding research is paramount to identify the right treatment for the right patient at the right time. Since these mediation analyses can be confounded by factors such as motivation or “healthy user” effects, randomized feature experiments (e.g., A/B testing) can provide stronger evidence on MoAs than observational mechanism analyses. Novel research techniques, such as micro-randomized controlled trials, may therefore be promising for DTx research^16^.

An example for clarification: A DTx for patients living with obesity aims to reduce weight by enabling patients to reflect on their diet via a photo-food diary. The MoA specifies that self-monitoring leads to heightened self-awareness regarding diet and enables a reflection on dietary improvements, which in turn leads to diet changes, which then lead to weight reduction (fundamental 1). Self-monitoring is operationalized as the number of photo-food logs in the first three weeks of the intervention (dose intake), the change in diet-related self-awareness is assessed via a survey (psychological dose realization) and the qualitative (i.e., healthier) change in logged meals (behavioral dose realization), and the clinical outcome is measured as change in relative weight (fundamentals 2 & 3). If several operationalizations or outcomes are tested, primary hypotheses should be specified in advance and robustness should be checked (e.g., via cross-validation).

Individual dose–response relationships are studied via responder analyses with self-monitoring (dose intake) and self-awareness and change in logged meals (dose realization) as predictors. Because dose intake and dose realization are not randomly assigned, these associations can be confounded and should not be over-interpreted as causal. Further, individual treatment trajectories and responses are studied with n-of-1-trials (fundamental 4). Applying the four fundamentals increases clarity, reproducibility, and may also assist developers to deliver effective DTx to patients.

This has implications for DTx research more broadly, requiring differentiation based on research aims and suitable methods: DTx mechanism of action research (e.g., DTx dose finding using causal forests in a large-scale RWD set) versus DTx efficacy evaluations (e.g., a prospective, randomized-control trial (RCT) comparing DTx treatment to a non-treated control group to inform a regulator’s decision on DTx reimbursement). Specifically, an RCT for a DTx efficacy evaluation provides group-based differences to assess the overall effect of the DTx. The RCT does not elucidate the mechanism of action of a DTx however, treating individuals’ dose–response trajectories as within-group variation. To understand the mechanism of action and individuals’ responses, alternative research designs - like micro-randomized controlled trials, sequential multiple-assignment randomized trials (SMART), or the multiphase optimisation strategy (MOST) - are likely to be more appropriate^17^.

## Discussion and avenues of future research

This conceptualization of MoA for DTx underscores the importance of clearly defined outcome measures that reflect the intended cognitive-affective or behavior changes. It also highlights both a large opportunity and critical need for DTx-generated RWD and DTx-derived real-world evidence (RWE)in advancing future research^18^. Special attention should be given to heterogeneous treatment effects and the substantial potential of DTx to not only document such heterogeneity, but also enable individualized treatment approaches.

In addition to comparisons against the standard of care, the focus of DTx research should be placed increasingly on comparisons *within* specific condition groups and mechanisms of action—or even at the individual level—given the diversity of therapeutic applications and patient needs. The growing availability of personalized therapies as well as complex interventions requires a broader and more fit-for-purpose evidence strategy. More data on observed MoA, both between groups in different therapies as well as within individuals over time (for example, through N-of-1 trials or adaptive clinical trial designs) is needed for a nuanced understanding of MoA for DTx. The user experience (UX) also plays an important role for DTx MoA. Two very similar treatment approaches (e.g., cognitive behavioral therapy for depression)– just distinguished by the user experience (e.g., frontend design, gamification etc.) – can lead to different clinical outcomes due to differences in consumed and/or realized doses. Fortunately, modern evidence generation approaches (especially harnessing RWE) beyond traditional randomized controlled trials can better leverage the potential of DTx^12,14,16^. This holds especially in times of fast-paced technological environments with continuous DTx advancements (e.g., UX evolution; incremental algorithm adjustments). This understanding therefore supports the development of fit-for-purpose DTx, tailored to specific clinical contexts.

Understanding the mechanism of action of DTx can yield better research, e.g., through properly selecting “placebo conditions” (i.e., placebo digital health applications)^19^. In pharmaceutical trials, placebo controls are used to isolate the drug’s biological effect from non-specific effects such as expectations or contextual influences. In contrast, many DTx exert their therapeutic effects through cognitive, emotional, or behavioral processes. As a result, placebo-controlled designs that aim to remove “psychological effects” are not directly transferable to DTx. Instead, evaluating DTx requires alternative and likely multi-dimensional control conditions that account for engagement, interaction, and digital context while selectively withholding the active therapeutic components of the intervention.

Moreover, DTx design can be maximized for clinical efficacy when MoA-related implications are observed: The design and refinement of impactful interventional elements (e.g., app features that drive cognitive-affective and behavior change in a clinically relevant way) should be prioritised. Accordingly, clinical impact assessments (validated measures of cognitive-affective and behavior change and their associated outcomes vs. mere engagement) can be used to evaluate the impact of interventional elements in a given DTx.

Pre-defining a recommended dose per DTx allows for per-protocol-analyses in clinical trials, which are based on participants’ adherence to a treatment regimen that is currently often ill-defined for DTx. The differentiation of intended, consumed, and realized dose (see Fig. 2) can help to define such treatment regimens as a starting point (e.g., a minimally important consumed dose), enabling analyses of dose–response associations, which may be relevant for future reimbursement policy.

Finally, with respect to adherence and reimbursement, a clearer understanding of mechanisms of action and use of RWE can support value-based payment frameworks that reflect the inherent heterogeneity of DTx treatments^14,20^. Germany provides a germane example: expected to start in 2026, performance-based reimbursement will be introduced to the DiGA pathway, although specific success measures still remain to be defined^15^. As described above, such approaches must be implemented thoughtfully and judiciously: basing reimbursement solely on general adherence metrics—such as time spent in the app or the number of logins—will fail to capture the diverse nature of DTx applications and their individualized treatment goals. This could result in poorly aligned incentives for manufacturers and lower-quality products for patients. Instead, success and effectiveness measures, including PROMs, should be tailored to individual applications or to groups of highly similar applications that share a MoA. These measures should then, in turn, be reflected in the clinical trial designs and outcomes used to validate the respective DTx intervention.

## Conclusion

When designing and evaluating DTx, their specific MoA requires thoughtful consideration and should directly impact study designs. Clear MoA definitions, specific operationalizations, and a focus on cognitive-affective and behavior change, i.e., dose realization and individual responses per DTx will drive clarity, comparability, and clinical efficacy gains in DTx research. Novel research methods that capitalize on the availability of large-scale and dense RWD-sets will be conducive to understanding how DTx work and will serve as a catalyst to the development of DTx with higher value to patients, HCPs, and the health care system more broadly.

## Data Availability

No datasets were generated or analyzed during the current study.
